# Supplementary material for: OsABT Is Involved in Abscisic Acid Signaling Pathway and Salt Tolerance of Roots at the Rice Seedling Stage
Source: Int J Mol Sci. 2022 Sep 13;23(18):10656. doi: 10.3390/ijms231810656 (PMC9504391; doi:10.3390/ijms231810656)
Supplement: Supplementary file 1 [file ijms-23-10656-s001.zip › ijms-1882538-supplementary.pdf]

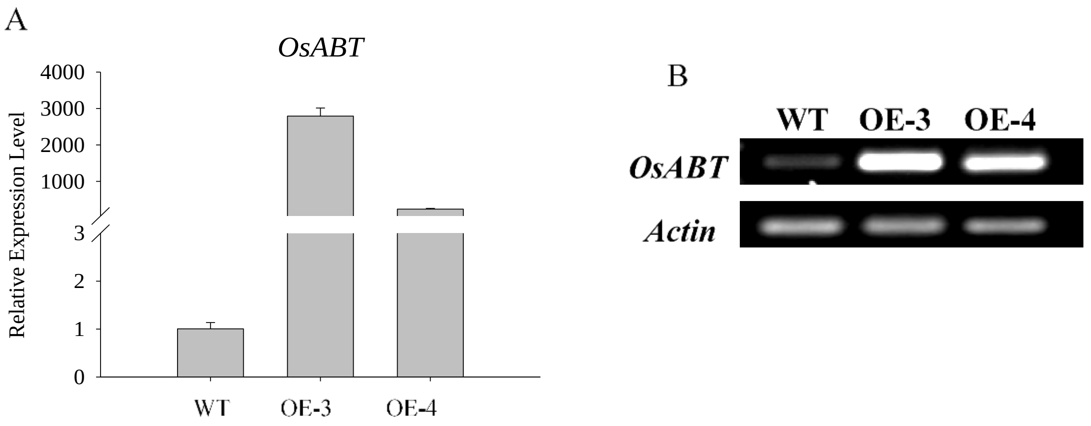

Figure S1. RT-PCR and qRT-PCR detection of *OsABT* overexpression lines. WT, wild type Nipponbare; OE-3 and OE-4, *OsABT* overexpression lines; Data are means ± SD of three independent experiments.

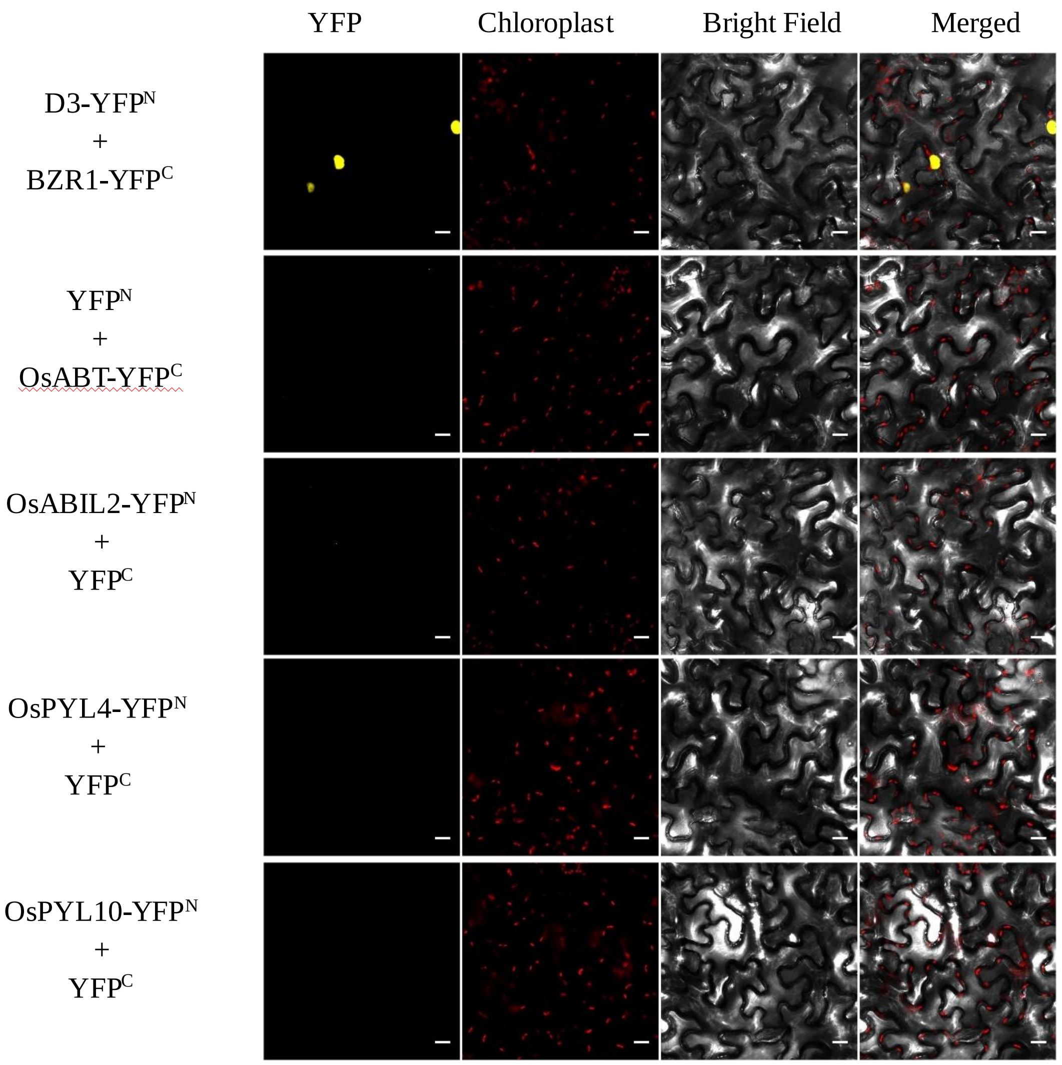

Figure S2. Bimolecular fluorescence complementation assay of the interactions between OsABT and OsABIL2, OsPYL4, OsPYL10 in *Nicotiana benthamiana* leaves. Scale bars: 50 μm.

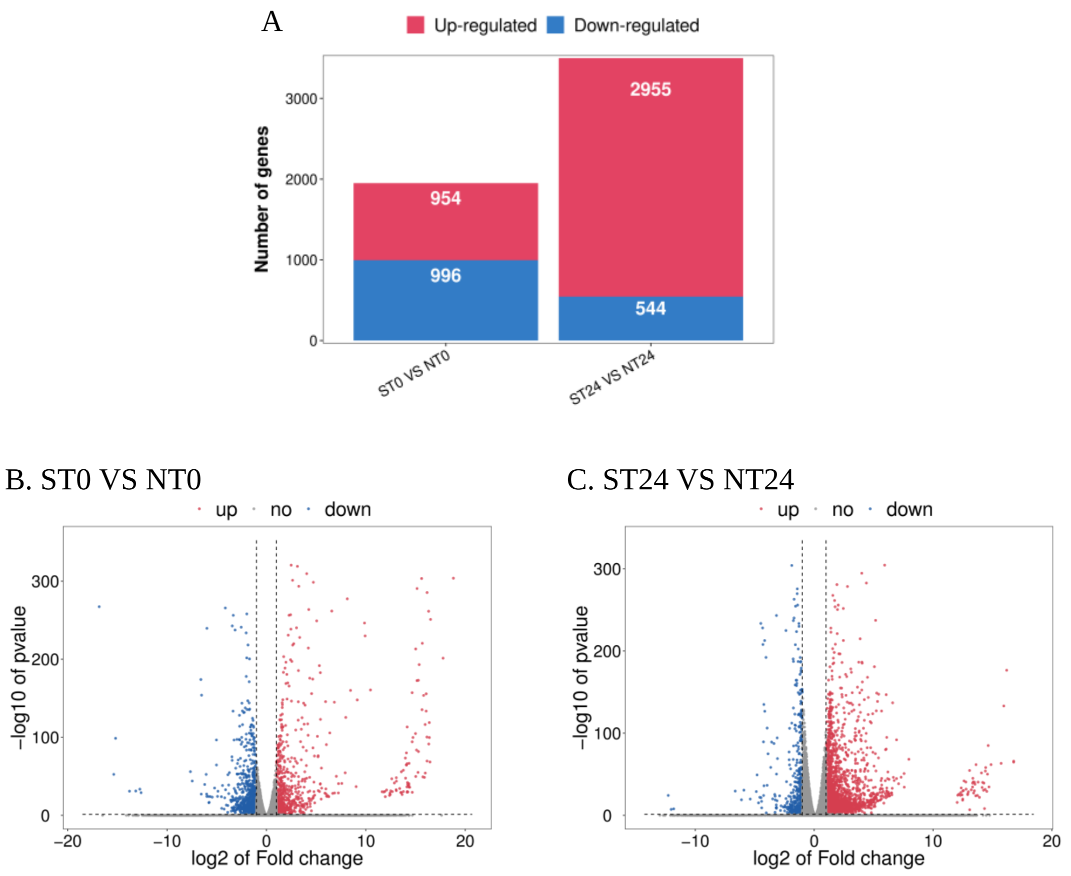

Figure S3. (**A**) Statistics of differentially expressed genes (DEGs). (**B**,**C**) The volcano map of DEGs in each comparison groups. The samples were named N representing Nipponbare, S representing *OsABT* overexpression line, T0 and T24 representing 200 mmol/L NaCl treatment for 0 h and 24 h.

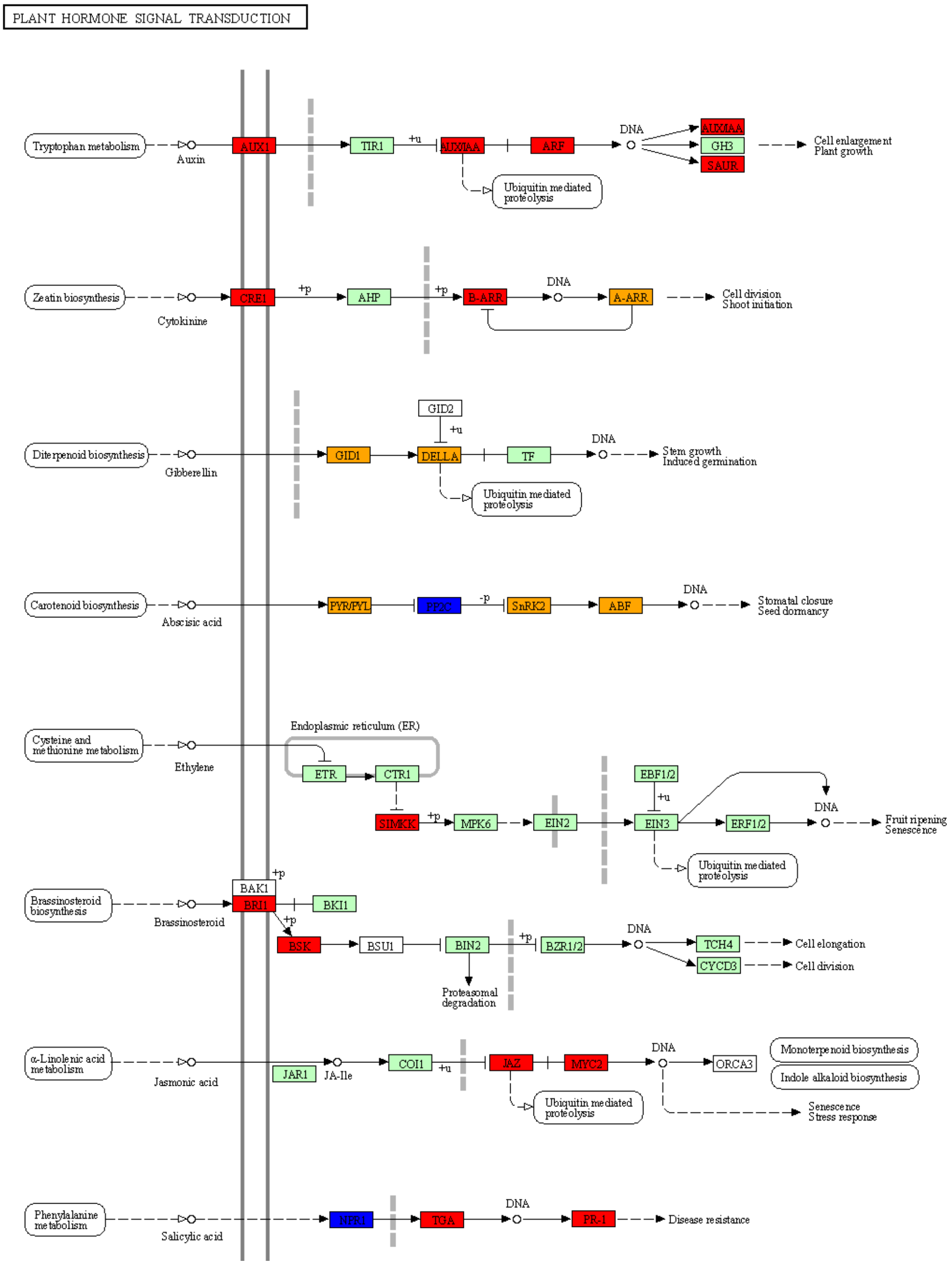

Figure S4. Plant hormone signal transduction between OE-3 and WT at 24 h of salt treatment. Red represents upregulated DEGs annotated to a ko node. Blue represents downregulated DEGs annotated to a ko node. Orange represents DEGs annotated to a ko node that are both upregulated and downregulated. Green indicates species-specific genes. Open circles indicate small molecule compounds. Solid arrows indicate the direction of biochemical reactions. Dashed arrows connect other related metabolic pathways.

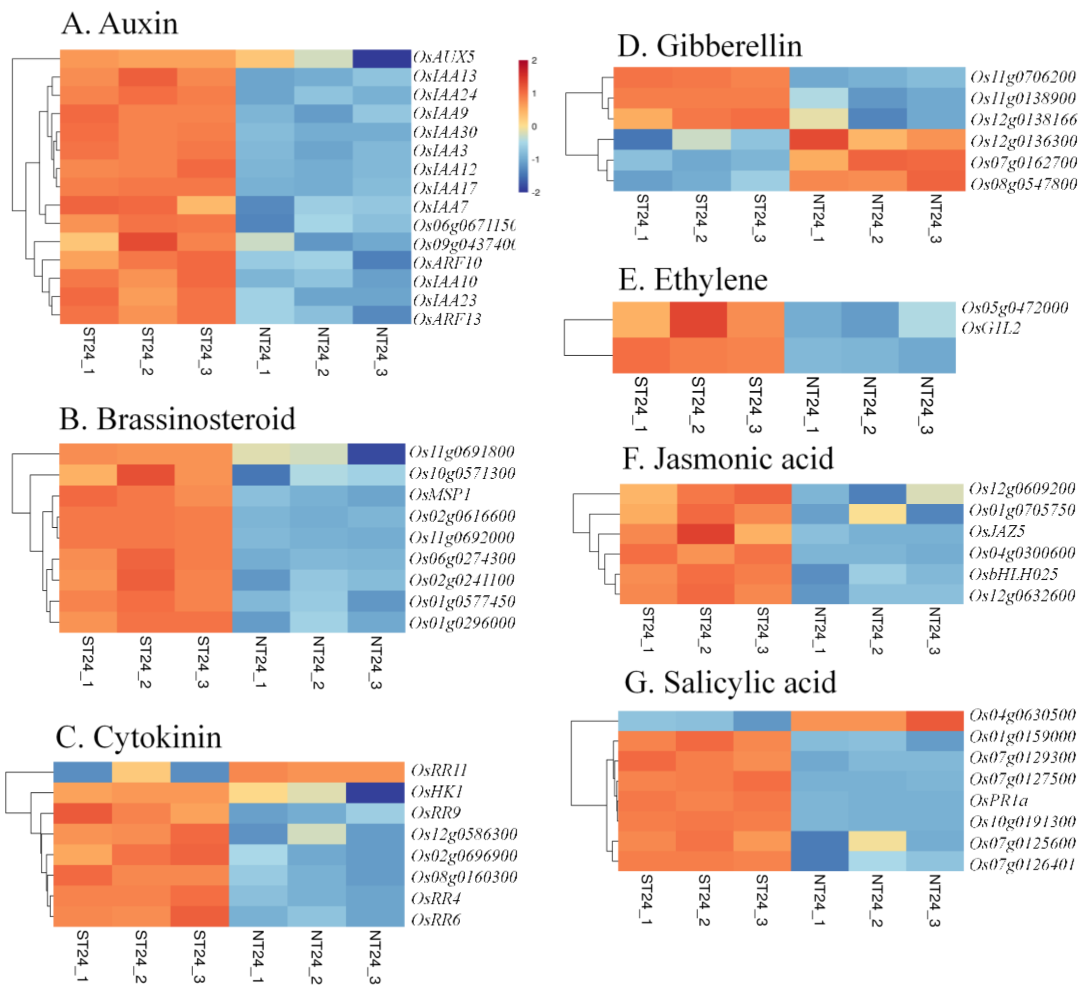

Figure S5 Cluster analysis of DEGs in other plant hormone signal transduction between OE-3 and WT at 24 h of salt treatment.

Table S1 Primers used for the qRT-PCR analysis and Y2H assay.

Primer name	Primer sequences (5'-3')
**OsABT-OE-F**	**GACTAGTTGATGAGAGATAGCGACGGCGA**
**OsABT-OE-R**	**CGAGCTCCTAGCGGAACGACGAGCTG**
**Actin-F**	**TGGCATCTCTCAGCACATTCC**
**Actin-R**	**TGCACAATGGATGGGTCAGA**
**OsABT-F**	**CCAAGACCGACACCCTGTAC**
**OsABT-R**	**TTCCACACCCTGATCTTGCC**
**OsSOS1-F**	**ACTTGGACGATGAGCCTGTG**
**OsSOS1-R**	**ATTTAGAAGCCGCACACGGA**
**OsHAK5-F**	**GATGTTGTTCTCAGTGCTGAGTG**
**OsHAK5-R**	**GTTCTGTGGTATGGTCAGGATTAGT**
**OsNCED3-F**	**CCCCTCCCAAACCATCCAAACCGA**
**OsNCED3-R**	**TGTGAGCATATCCTGGCGTCGTGA**
**OsNCED4-F**	**ACGGCGGAGAAGTTCATC**
**OsNCED4-R**	**TGGACGAAGCACAGCAC**
**OsABA8ox2-F**	**GGCGAGCATAATCTCCTTCA**
**OsABA8ox2-R**	**CCCTTTGGAATCAGGAAACC**
**OsPYL5-F**	**CATCCTCAGCGTCAAGTTCG**
**OsPYL5-R**	**TCACAAGCGTCCCTGGTCT**
**OsPYL9-F**	**GGCGGAGGACACCAGGAT**
**OsPYL9-R**	**CGACGATTTATTGACGAGG**
**OsABIL2-F**	**CCTCCTTCCGCCTTCCAG**
**OsABIL2-R**	**CGTCGTCCTCCGCTCTAC**
**OsLEA3-F**	**CAACAGGCGAGTGAGCAGGT**
**OsLEA3-R**	**GGCAGAGGTGTCCTTGTTGG**
**Rab16A-F**	**GCACACCACAGCAAGAG**
**Rab16A-R**	**GGTGCTCCATCCTGCTTA**
**pBD-OsABT-F**	**ttgactgtatcgccggaattcATGAGAGATAGCGACGGCGA**
**pBD-OsABT-R**	**gctcgaggcccgggcgaattcCTAGCGGAACGACGAGCTGC**
**pAD-OsABIL2-F**	**tcctctgctagcagagaattcATGGAGGACCTCGCCCTG**
**pAD-OsABIL2-R**	**aagcattagagaattgaattcTCATGCTTTGCTCTTGAACTTCC**
**pAD-OsPYL4-F**	**tcctctgctagcagagaattcATGCCGTGCATCCCGGCG**
**pAD-OsPYL4-R**	**aagcattagagaattgaattcTCACGAGCCGGCGGCCCT**
**pAD-OsPYL10-F**	**tcctctgctagcagagaattcATGGAGCAGCAGGAGGAAGTG**
**pAD-OsPYL10-R**	**aagcattagagaattgaattcCTATTCCGCCGCCGCCGG**
